# THE EFFECTS OF COVID-19 ON THE MIGRATION OF HOME HEALTHCARE WORKER

**DOI:** 10.1093/geroni/igac059.373

**Published:** 2022-12-20

**Authors:** Pamela Monaghan-Geernaert

**Affiliations:** Northern State University, Aberdeen, South Dakota, United States

## Abstract

Home health caregivers provide a vital role in allowing people to age in place. Women, and in particular immigrant women, have become the largest provider of home health care in Western Industrialized Countries. Global push-pull factors affecting caregivers were greatly disrupted with the emergence of the global pandemic – COVID-19. The pandemic highlighted how fragile the system is and how the easily the ‘grey economy’ can be compromised. This research looks at border access, immigration restrictions and other factors which impeded the migration of caregivers. Poor countries suffered greatly with restrictions on exporting women who use cross national moves as a means to generate income for families in their homeland, and rich countries faced extreme shortages. The results were devastating for all parties – caregivers and care recipients alike.

